# Supplementary material for: Ensembled deep learning model outperforms human experts in diagnosing biliary atresia from sonographic gallbladder images
Source: Nat Commun. 2021 Feb 24;12:1259. doi: 10.1038/s41467-021-21466-z (PMC7904842; doi:10.1038/s41467-021-21466-z)
Supplement: Supplementary file 1 — Supplementary Information [file 41467_2021_21466_MOESM1_ESM.pdf]

Ensembled Deep Learning Model Outperforms Human Experts in Diagnosing Biliary Atresia from Sonographic Gallbladder Images

Wenying Zhou, Yang Yang, Cheng Yu, Juxian Liu, Xingxing Duan, Zongjie Weng, Dan Chen, Qianhong Liang, Fang Qing, Jiaojiao Zhou, Hao Ju, Zhenhua Luo, Weihao Guo, Xiaoyan Ma, Xiaoyan Xie, Ruixuan Wang, Luyao Zhou

Supplementary methods

Supplementary Note 1: Images from human subjects

Ultrasonic gallbladder images used as training cohort were collected from the First Affiliated Hospital, Sun Yat-sen University, Hunan Children's Hospital, Fujian Provincial Maternity and Children's Hospital, Affiliated Hospital of Fujian Medical University, Guangdong Women and Children' Hospital, and Shengjing Hospital of China Medical University. Ultrasonic gallbladder images used as external validation cohort were collected from Union Hospital, Tongji Medical College of Huazhong University of Science and Technology, West China Hospital of Sichuan University, Hexian Memorial Affiliated Hospital of Southern Medical University, the First People's Hospital of Foshan, Tongji Hospital, Tongji Medical College, Huazhong University of Science and Technology, and Sanya City Womenfolk and Infant Health Care Hospital.

Supplementary Note 2: The SE-ResNet model structure

Multiple CNN model architectures have been proposed in the past several years, e.g., VggNet [1], ResNet [2], SE-ResNet [3] and SENet [3], with both SE-ResNet and SENet showing superior performance

on natural images (e.g., ImageNet) compared to most other model architectures. Considering that SENet was much more computationally expensive than SE-ResNet during training, we adopted SE-ResNet as the CNN architecture for intelligent analysis of BA in this study. The adopted SE-ResNet mainly consists of 50 residual convolutional units, with each unit followed by a squeeze-and-excitation (SE) block (Supplementary Fig. 4). Each residual unit is composed of three convolutional layers and a skip connection from the input of the first layer to the output of the last (third) layer. Each convolutional layer contains multiple mathematical convolution operations between the input of the layer and the convolutional kernels in the layer. Batch normalization and a rectified linear unit (ReLU) are also part of each convolutional layer by default. The SE block is a three-layer fully connected sub-network inserted to the end of each residual unit to adaptively adjust the importance (or "excitation") of each output channel of the residual unit by considering (or "squeezing") the global visual information of the input image. With this SE block, relevant visual features would become more excited, while irrelevant visual features would be suppressed to a large extent, therefore making the model focus on more relevant information during prediction. Besides, the last fully connection layer was adapted for our binary classification task.

Supplementary Note 3: Training the SE-ResNet

Before training each SE-ResNet, all images from the training cohort were pre-processed as follows. First, from each original image, six new images were obtained by randomly cropping around the provided bounding box, with each new image slightly larger than and containing the entire gallbladder region. Then, each new image was rescaled to a square image of the size 224-by-224 pixels. Each single-channel grayscale image became a three-channel image by duplicating the original single

channel three times. The mean and standard deviation of pixel intensities were computed over all cropped images, which were then used to normalize each pixel in all rescaled images. The normalization method was consistent with the pretrained model used. The pre-trained model we used comes from one of the most commonly used repositories (https://github.com/Cadene/pretrained-models.pytorch) on the github. Each normalized image will be used as an input to the SE-ResNet for model training.

Training a SE-ResNet was an iterative process. At each iteration, a batch of (normalized) images were respectively input to the SE-ResNet, and the scalar output of the SE-ResNet would be compared to the expected output ("1" if the class of the input image was BA, and "0" for non-BA). Training a deep learning model is actually to update the parameters of the model such that the real outputs are as close to the expected outputs as possible for each batch of input images, where the model parameters mainly consist of the elements in each convolution kernel in each convolutional layer and the edge weights in each fully connect layer. The differences between the real outputs and the expected outputs are measured by a loss function called cross-entropy loss, which is a mathematical function of the model parameters. In other words, model training is to search for the best set of model parameter values such that the cross-entropy loss is minimum with the training images as input to the model. Mini-batch stochastic gradient descent (SGD) is one of the widely used methods to find the best model parameters over iterations, and was used for all model trainings in this study, where the batch size (i.e., the number of the images) was set to 16, and the learning rate of the SGD method was initially set to 0.01 and divided by 10 after every 35 epochs. Each epoch consisted of a sequence of training iterations through which all training images were fed into the model once. The maximum number of epochs was set to 210 before which each model has been well trained without much change in model parameters.

To improve the generalizability of each SE-ResNet, besides the ensembled learning mentioned in the main text, a few effective tricks were also applied for model training. First, considering that there were fewer images for the BA class than for the non-BA class, the cross-entropy loss was slightly modified using the well-known cost-sensitive learning [4] to improve the influence of BA each image. Second, a pre-trained SE-ResNet based on the large-scale natural image dataset ImageNet was used to initialize the model parameters, because such initialization has been experimentally proven effective in improving the classification performance particularly for new classification tasks with relatively small training dataset [5]. The last fully connection layer in the SE-ResNet was randomly initialized for our binary classification task, as commonly adopted when using a pre-trained model. Third, the drop-out operation was only applied to the last fully connect layer of the SE-ResNet, which has shown to reduce the potential inter-dependence between neurons in the network and therefore improve the generalizability of the model [6]. The rate of dropout was set to 0.2 for all training period in this study. In addition, besides the random crop mentioned above, the horizontal flipping of each training image at a probability 0.5 was also used as part of data augmentation during model training.

Supplementary Note 4: Alternative training strategies and CNN backbones

The proposed ensemble model was empirically compared with a few alternatives, including (1) an individual CNN model trained with the entire training dataset (Tables 6-9, fourth row), (2) the general ensemble model (of 5 and 10 individual models respectively) where each individual model was trained with the entire training dataset (Tables 6-9, last two rows), (3) the ensemble model with the proposed k-fold ensemble strategy, but with different k values (k= 3 and 10 respectively, Tables 6-9, second and third row), (4) the individual and various ensemble models with different CNN backbones (Tables 6-

9), and (5) the ensemble of five different individual models with different backbones (SE_ResNet-152, DenseNet-201, EfficientNet-B7, ResNet-152 and Vgg-19). When using the entire training dataset to train an individual model, each individual model was trained to the maximum number of epochs (i.e., 210 epochs), with consistent observation of training convergence.

From Tables 6-9, it could be observed that the proposed ensemble model (first row) consistently performed better (in AUCs) than the either the individual model (four row) or the ensemble of multiple individual models (last two rows) where each individual model was trained with the entire training dataset, no matter which model backbone was used (Tables 6-9). While the proposed ensemble model was based on 5-fold cross validation, different number of folds could be used. In this study, the diagnostic performance of the ensemble models trained with 3-fold and 10-fold cross-validation (second and third row) was either lower or comparable to that of the model based on the 5-fold cross-validation. The number of folds in the proposed ensemble strategy could be considered as a hyperparameter and experimentally determined for any specific application. In addition, when combining five different individual models (SE_ResNet-152, DenseNet-201, EfficientNet-B7, ResNet-152 and VggNet-19) to form an ensemble model, the ensemble model yielded a sensitivity 80.5% and a specificity of 93.9% (AUC 0.925) when trained by 5-fold cross validation, and yielded a sensitivity 72.9 % and a specificity of 97.0% (AUC 0.901) when trained with the entire dataset for each individual model. These two AUCs were clearly lower than the result (AUC 0.942) obtained by the ensemble model of five SE_ResNet-152 models based on the proposed ensemble strategy (Table 6, first row).

Supplementary Note 5: Libraries

The image processing libraries used for training model included PyTorch (1.5.1), torchvision

(0.6.1), NumPy (1.17.0), scikit-learn (0.21.3), TensorboardX (1.8), PIL (7.1.2), tqdm (4.32.1), SimpleITK (1.2.0), pandas (0.25.0), Matplotlib (3.1.1), pretrained-models (0.7.4, https://github.com/Cadene/pretrained-models.pytorch), and EfficientNet-PyTorch (0.6.3, https://github.com/lukemelas/EfficientNet-PyTorch).

Supplementary Tables

Supplementary Table 1. Comparison of the diagnostic performances at the image level between the model and two radiologists under various scanning conditions on the internal cross-validation dataset.

Condition	Training#	Test#		AUC	Sensitivity (%)	Specificity (%)	P value*
Frequency	< 14 (n=1467)	⩾14 (n=2238)	AI model	0.931 (0.919 - 0.941)	83.5 (79.8 - 86.8)	95.6 (94.5 - 96.5)	-
			Expert A	0.773 (0.755 - 0.790)	93.9 (91.3 - 95.9)	60.6 (58.3 - 62.9)	<.001
			Expert B	0.828 (0.811 - 0.843)	73.5 (69.3 - 77.5)	92.0 (90.6 - 93.2)	<.001
	⩾14 (n=2238)	< 14 (n=1467)	AI model	0.835 (0.815 - 0.853)	64.2 (59.7 - 68.6)	91.4 (89.5 - 93.1)	-
			Expert A	0.676 (0.652 - 0.700)	93.8 (91.1 - 95.8)	41.5 (38.4 - 44.6)	<.001
			Expert B	0.842 (0.822 - 0.860)	79.1 (75.1 - 82.7)	89.2 (87.1 - 91.1)	.677
Screening time	⩽2018 (n=2477)	>2018 (n=1228)	AI model	0.900 (0.882 - 0.916)	90.9 (88.0 - 93.3)	77.8 (74.6 - 80.8)	-
			Expert A	0.641 (0.613 - 0.668)	96.2 (94.1 - 97.7)	32.0 (28.7 - 35.5)	<.001
			Expert B	0.810 (0.786 - 0.831)	79.8 (75.9 - 83.2)	82.2 (79.2 - 84.9)	<.001
	>2018 (n=1228)	⩽2018 (n=2477)	AI model	0.832 (0.817 - 0.847)	68.0 (63.3 - 72.4)	87.7 (86.2 - 89.1)	-
			Expert A	0.763 (0.746 - 0.780)	91.2 (88.1 - 93.7)	61.5 (88.1 - 93.7)	<.001
			Expert B	0.833 (0.818 - 0.847)	72.4 (67.9 - 76.6)	94.2 (93.1 - 95.2)	0.964
Machine	Mindray + the others (n=2332)	Supersonic (n=1373)	AI model	0.807 (0.784 - 0.829)	85.4 (82.3 - 88.0)	66.1 (62.1 - 69.9)	-
			Expert A	0.585 (0.556 - 0.613)	95.5 (93.5 - 97.0)	21.5 (18.2 - 25.0)	<.001
			Expert B	0.760 (0.735 - 0.784)	81.3 (78.0 - 84.3)	70.8 (66.9 - 74.4)	.007
	Mindray + Supersonic (n=2492)	The others (n=1213)	AI model	0.787 (0.762 - 0.811)	62.1 (55.9 - 68.0)	87.8 (85.4 - 89.9)	-
			Expert A	0.722 (0.694 - 0.748)	92.3 (88.4 - 95.3)	52.0 (48.6 - 55.4)	.004
			Expert B	0.817 (0.793 - 0.840)	70.1 (64.2 - 75.6)	93.4 (91.5 - 94.9)	.199
	Supersonic + the others (n=2586)	Mindray (n=1119)	AI model	0.950 (0.937 - 0.960)	83.7 (69.3 - 93.2)	99.3 (98.6 - 99.6)	-
			Expert A	0.741 (0.717 - 0.764)	79.1 (64.0 - 90.0)	69.2 (66.6 - 71.6)	<.001
			Expert B	0.702 (0.677 - 0.726)	41.9 (27.0 - 57.9)	98.5 (97.7 - 99.1)	<.001

Note: #Numbers of training and test images are included in brackets in the second and third columns. 95% confidence intervals are included in brackets in other relevant columns.

*The P values were from the comparison between the AUC of the ensemble deep learning model and the AUCs of two human experts. Differences between various AUCs were compared using a Delong test.

'AI', artificial intelligence; 'AUC', area under receiver operating characteristic curve.

Supplementary Table 2. Comparison of the diagnostic performances at the patient level between the model and two radiologists under various scanning conditions on the internal cross-validation dataset.

Condition	Training#	Test#		AUC	Sensitivity (%)	Specificity (%)	P value*
Frequency	< 14 (n=624)	⩾14 (n=517)	AI model	0.921 (0.902 - 0.945)	83.6 (77.3 - 88.7)	94.5 (92.0 - 96.3)	-
			Expert A	0.739 (0.704 - 0.772)	90.4 (85.1 - 94.3)	57.5 (53.0 - 61.9)	<.001
			Expert B	0.804 (0.772 - 0.833)	63.8 (56.3 - 70.9)	96.9 (95.0 - 98.3)	<.001
	⩾14 (n=517)	< 14 (n=624)	AI model	0.828 (0.796 - 0.856)	69.2 (62.7 - 75.2)	86.3 (82.5 - 89.5)	-
			Expert A	0.715 (0.678 - 0.750)	89.3 (84.5 - 93.0)	53.8 (48.7 - 58.7)	.030
			Expert B	0.821 (0.789 - 0.851)	68.8 (62.2 - 74.8)	95.5 (93.0 - 97.3)	.098
Screening time	⩽2018 (n=881)	>2018 (n=260)	AI model	0.911 (0.888 - 0.934)	93.9 (87.8 - 97.5)	78.9 (71.4 - 85.2)	-
			Expert A	0.643 (0.582 - 0.701)	91.2 (84.5 - 95.7)	37.4 (29.6 - 45.8)	<.001
			Expert B	0.811 (0.759 - 0.857)	68.4 (59.1 - 76.8)	93.9 (88.7 - 97.2)	.122
	>2018 (n=260)	⩽2018 (n=881)	AI model	0.820 (0.791 - 0.852)	72.2 (65.7 - 78.1)	82.4 (79.3 - 85.2)	-
			Expert A	0.757 (0.727 - 0.785)	89.4 (84.5 - 93.1)	62.0 (58.1 - 65.7)	.496
			Expert B	0.809 (0.781 - 0.834)	64.4 (57.6 - 70.7)	97.4 (95.9 - 98.5)	.169
Machine	Mindray + the others (n=828)	Supersonic (n=313)	AI model	0.805 (0.769 - 0.833)	87.8 (82.3 - 92.0)	63.9 (56.0 - 71.2)	-
			Expert A	0.619 (0.567 - 0.670)	91.3 (86.5 - 94.9)	32.5 (25.5 - 40.2)	<.001
			Expert B	0.789 (0.743 - 0.830)	70.4 (63.5 - 76.7)	87.4 (81.3 - 92.0)	.345
	Mindray + Supersonic (n=779)	The others (n=362)	AI model	0.761 (0.728 - 0.796)	63.1 (53.9 - 71.7)	83.0 (78.6 - 86.8)	-
			Expert A	0.766 (0.725 - 0.804)	89.3 (82.5 - 94.2)	63.9 (58.6 - 68.9)	.303
			Expert B	0.803 (0.764 - 0.838)	61.5 (52.2 - 70.1)	99.1 (97.5 - 99.8)	.053
	Supersonic + the others (n=675)	Mindray (n=466)	AI model	0.954 (0.935 - 0.969)	83.3 (51.6 - 97.9)	99.0 (97.1 - 99.8)	-
			Expert A	0.694 (0.640 - 0.745)	75.0 (42.8 - 94.5)	63.8 (58.1 - 69.2)	.015
			Expert B	0.663 (0.608 - 0.716)	33.3 (9.9 - 65.1)	99.3 (97.6 - 99.9)	.011

Note: #Numbers of training and test patients are included in brackets in the second and third columns. 95% confidence intervals are included in brackets in other relevant columns.

*The P values were from the comparison between the AUC of the ensemble deep learning model and the AUCs of two human experts. Differences between various AUCs were compared using a Delong test.

'AI', artificial intelligence; 'AUC', area under receiver operating characteristic curve.

Supplementary Table 3. Consistency assessment of regions of interest between one individual model within the ensemble deep learning model and human radiologists on the external validation dataset.

AI Model	Real diagnosis	Regions of interest		
		Consistent	Inconsistent	Total
Correctly diagnosed	Biliary atresia	206 (98.6%)	3 (1.4%)	209
	Non biliary atresia	566 (99.8%)	1 (0.2%)	567
	Total	772 (99.5%)	4 (0.5%)	776
Incorrectly diagnosed	Biliary atresia	27 (100%)	0 (0%)	27
	Non biliary atresia	38 (100%)	0 (0%)	38
	Total	65 (100%)	0 (0%)	65

Note: Data are numbers of images and the percentage in parentheses.

The attended regions obtained by the class activation map during diagnosis by the individual model which was within the ensembled deep learning model but had strong activation and the same classification result as that of the ensemble deep learning model.

'AI', artificial intelligence.

Supplementary Table 4. Statistics of subject information in this study.

Characteristic	Training dataset /Internal validation dataset			External validation dataset		
	Infants with jaundice		Infants without jaundice	Infants with jaundice		Infants without jaundice
	BA group	Non-BA group		BA group	Non-BA group	
Gender						
N (Missing)	330 (0)	531 (0)	280 (0)	102 (0)	137(0)	59 (0)
Male (%)	180 (54.5)	352 (66.3)	182 (65)	50 (49)	84 (61.3)	36 (61)
Female (%)	150 (45.5)	179 (33.7)	98 (35)	52 (51)	53 (38.7)	23 (39)
Age (days)						
N (Missing)	330 (0)	531 (0)	280 (0)	102 (0)	137(0)	59 (0)
Mean ± SD	60.0 ± 21.2	58.8 ± 24.7	52.8 ± 24.6	57.9 ± 26.0	57.4 ± 20.0	33.0 ± 26.9
Min, Max	7.0, 141.0	5.0, 143.0	3.0, 145.0	18.0, 146.0	10.0, 120.0	11.0, 136.0
TB (mmol/L)			-			-
N (Missing)	328 (2)	496 (35)		94 (8)	137 (0)	
Median	166.8	136.3		173.9	116.6	
Q1, Q3	141.3, 201.9	98.8, 193.4		138.9, 225.0	76.2, 165.1	
DB (mmol/L)			-			-
N (Missing)	328 (2)	496 (35)		94 (8)	137 (0)	
Median	106.3	85.2		117.4	73.8	
Q1, Q3	86.4, 126.0	61.3, 119.5		96.9, 150.2	48.6, 99.4	
IB (mmol/L)			-			-
N (Missing)	328 (2)	496 (35)		94 (8)	137 (0)	
Median	61.1	45.8		51.1	44.0	
Q1, Q3	40.3, 83.6	27.6, 75.9		28.9, 78.6	25.9, 70.3	
ALT (U/L)			-			-
N (Missing)	296 (34)	471 (60)		65 (37)	74 (63)	
Median	136.5	111.0		137.3	85.2	
Q1, Q3	90.3, 225.7	66.1, 192.8		96.0, 214.0	49.3, 147.5	
AST (U/L)			-			-
N (Missing)	302 (28)	445 (86)		65 (37)	74 (63)	
Median	189.5	109.4		133.7	77.5	
Q1, Q3	135.4, 282.8	57.3, 191.0		85.5, 195.3	38.9, 159.0	

Note: ‘N’, number of subjects; ‘Q’, quartile; ‘BA’, biliary atresia; ‘TB’, total bilirubin; ‘DB’, direct bilirubin; ‘IB’, indirect bilirubin; ‘ALT’, alanine aminotransferase; ‘AST’, aspartate aminotransferase.

Supplementary Table 5. The number of -patients and -images in training (internal validation) dataset and external validation dataset.

Dataset	Hospitals*	Infants with jaundice		Infants without jaundice	Total
		BA group	Non-BA group		
Training	5	925 (330)	2149 (531)	631 (280)	3705 (1141)
External validation	6	236 (102)	467 (137)	138 (59)	841 (298)

Note: Data are number of images and number of patients in brackets in the last four columns.

*the number of the hospitals providing patient data. Training dataset and external validation dataset were from different hospitals.

'BA', biliary atresia

Supplementary Table 6. Comparison of the diagnostic performances at the image level on SE_ResNet-152 using different ensemble methods on the external validation dataset.

Ensemble	Number of Individual models	Training data for each individual model	AUC	Sensitivity (%)	Specificity (%)	Accuracy (%)	PPV (%)	NPV (%)	P value*
✓	5	Part	0.942 (0.924, 0.957)	88.6 (83.8, 92.3)	93.7 (91.5, 95.5)	92.3	84.6	95.5	-
✓	3	Part	0.935 (0.916, 0.951)	85.6 (80.5, 89.8)	93.7 (91.5, 95.5)	91.4	84.2	94.3	.246
✓	10	Part	0.938 (0.920, 0.954)	88.6 (83.8, 92.3)	94.1 (91.9, 95.8)	92.5	85.3	95.5	.448
×	1	All	0.930 (0.911, 0.946)	74.2 (68.1, 79.6)	95.4 (93.4, 96.9)	89.4	86.2	90.4	.299
✓	5	All	0.898 (0.876, 0.918)	74.58 (68.5, 80.0)	97.0 (95.3, 98.2)	90.7	90.7	90.7	<.001
✓	10	All	0.890 (0.867, 0.910)	75.4 (69.4, 80.8)	97.0 (95.3, 98.2)	91.0	90.8	91.0	<.001

Note: 95% confidence intervals are included in brackets.

*The P values were from the comparison between the AUC of the proposed 5-fold ensemble deep learning model (‘Part’) and the AUCs of the others. Differences between various AUCs were compared using a Delong test.

‘AUC’, area under receiver operating characteristic curve; ‘PPV’, Positive predictive value; ‘NPV’, Negative predictive value.

Supplementary Table 7. Comparison of the diagnostic performances at the image level on DenseNet-201 using different ensemble methods on the external validation dataset.

Ensemble	Number of Individual models	Training data for each individual model	AUC	Sensitivity (%)	Specificity (%)	Accuracy (%)	PPV (%)	NPV (%)	P value*
✓	5	Part	0.893 (0.870, 0.913)	80.9 (75.3, 85.7)	83.8 (80.6, 86.6)	83.0	66.1	91.8	-
✓	3	Part	0.888 (0.864, 0.908)	80.5 (74.9, 85.4)	84.3 (81.1, 87.1)	83.2	66.7	91.7	.305
✓	10	Part	0.874 (0.850, 0.896)	79.2 (73.5, 84.2)	85.6 (82.6, 88.3)	83.8	68.2	91.4	.016
×	1	All	0.878 (0.854, 0.899)	69.5 (63.2, 75.3)	95.9 (94.0, 97.3)	88.5	86.8	89.0	.198
✓	5	All	0.882 (0.858, 0.903)	72.5 (66.3, 78.1)	95.9 (94.0, 97.3)	89.3	87.2	89.9	.271
✓	10	All	0.873 (0.848, 0.894)	73.7 (67.6, 79.2)	95.5 (93.6, 97.0)	89.4	86.6	90.3	.056

Note: 95% confidence intervals are included in brackets.

*The P values were from the comparison between the AUC of the proposed 5-fold ensemble deep learning model (‘Part’) and the AUCs of the others. Differences between various AUCs were compared using a Delong test.

‘AUC’, area under receiver operating characteristic curve; ‘PPV’, Positive predictive value; ‘NPV’, Negative predictive value.

Supplementary Table 8. Comparison of the diagnostic performances at the image level on EfficientNet-B7 using different ensemble methods on the external validation dataset.

Ensemble	Number of Individual models	Training data for each individual model	AUC	Sensitivity (%)	Specificity (%)	Accuracy (%)	PPV (%)	NPV (%)	P value*
✓	5	Part	0.945 (0.927, 0.959)	90.7 (86.2, 94.1)	89.9 (87.2, 92.2)	90.1	77.8	96.1	-
✓	3	Part	0.949 (0.932, 0.963)	89.8 (85.2, 93.4)	89.6 (86.9, 91.9)	89.7	77.1	95.8	.356
✓	10	Part	0.928 (0.909, 0.945)	89.0 (84.3, 92.7)	91.1 (88.5, 93.2)	90.5	79.5	95.5	.002
×	1	All	0.889 (0.866, 0.909)	74.2 (68.1, 79.6)	95.0 (93.0, 96.6)	89.2	85.4	90.4	<.001
✓	5	All	0.903 (0.881, 0.922)	0.767 (70.8, 81.9)	94.9 (92.8, 96.5)	89.8	85.4	91.3	.002
✓	10	All	0.895 (0.872, 0.915)	79.2 (73.5, 84.2)	96.0 (94.2, 97.4)	91.3	88.6	92.2	<.001

Note: 95% confidence intervals are included in brackets.

*The P values were from the comparison between the AUC of the proposed 5-fold ensemble deep learning model (‘Part’) and the AUCs of the others. Differences between various AUCs were compared using a Delong test.

‘AUC’, area under receiver operating characteristic curve; ‘PPV’, Positive predictive value; ‘NPV’, Negative predictive value.

Supplementary Table 9. Comparison of the diagnostic performances at the image level on ResNet-152 using different ensemble methods on the external validation dataset.

Ensemble	Number of Individual models	Training data for each individual model	AUC	Sensitivity (%)	Specificity (%)	Accuracy (%)	PPV (%)	NPV (%)	P value*
✓	5	Part	0.904 (0.882, 0.923)	82.2 (76.7, 86.9)	90.6 (88.0, 92.8)	88.2	77.3	92.9	-
✓	3	Part	0.908 (0.886, 0.927)	80.5 (74.9, 85.4)	88.6 (85.8, 91.0)	86.3	73.4	92.1	.671
✓	10	Part	0.906 (0.884, 0.924)	83.1 (77.6, 87.6)	89.9 (87.2, 92.2)	88.0	76.3	93.2	.772
×	1	All	0.867 (0.842, 0.889)	70.3 (64.1, 76.1)	95.5 (93.6, 97.0)	88.5	86.0	89.2	.020
✓	5	All	0.887 (0.864, 0.908)	71.6 (65.4, 77.3)	95.7 (93.8, 97.2)	88.9	86.7	89.6	.200
✓	10	All	0.871 (0.846, 0.893)	72.0 (65.8, 77.7)	96.0 (94.2, 97.4)	89.3	87.6	89.8	.021

Note: 95% confidence intervals are included in brackets.

*The P values were from the comparison between the AUC of the proposed 5-fold ensemble deep learning model (‘Part’) and the AUCs of the others. Differences between various AUCs were compared using a Delong test.

‘AUC’, area under receiver operating characteristic curve; ‘PPV’, Positive predictive value; ‘NPV’, Negative predictive value.

Supplementary Figures

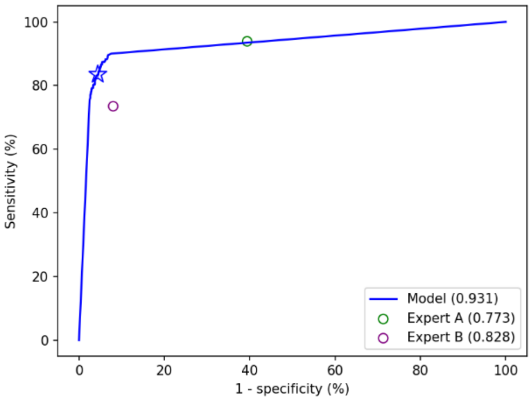

a

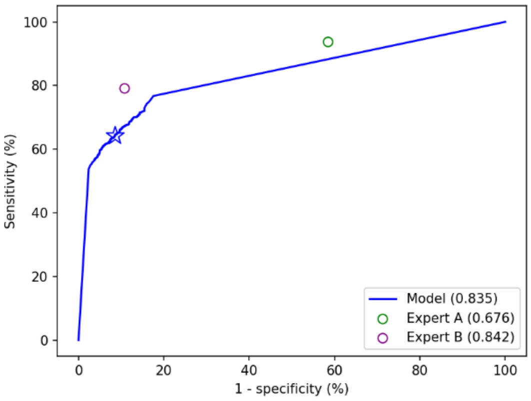

b

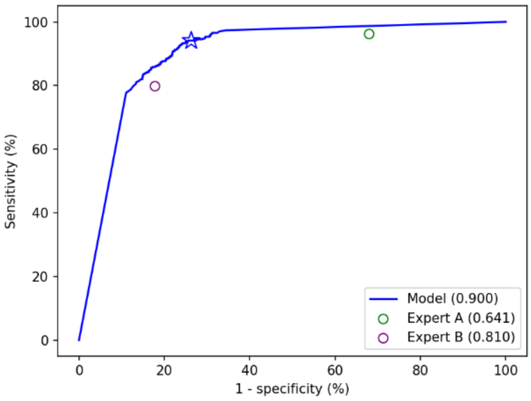

c

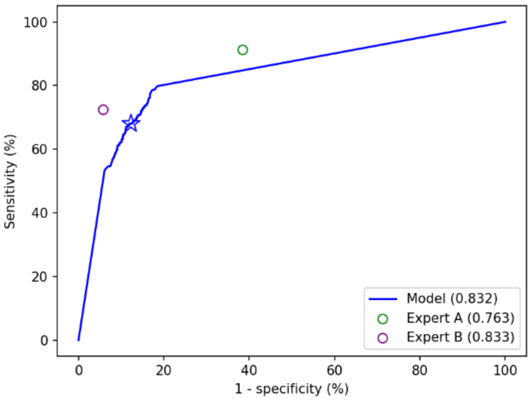

d

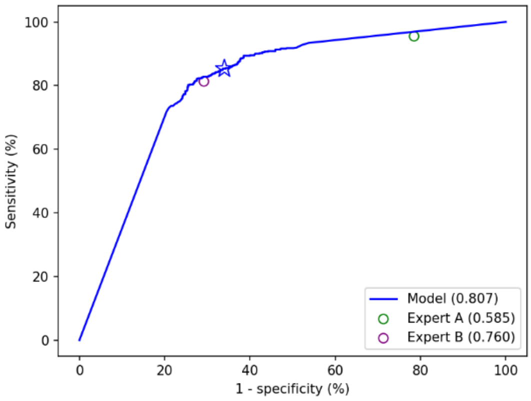

e

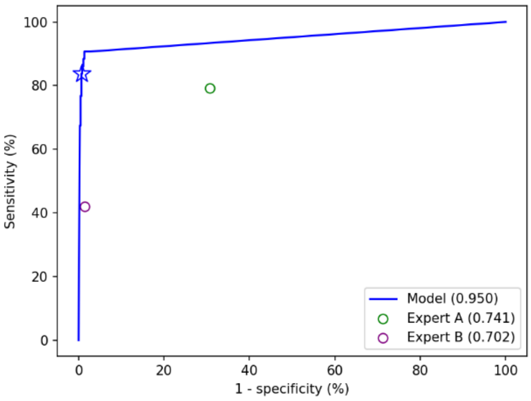

f

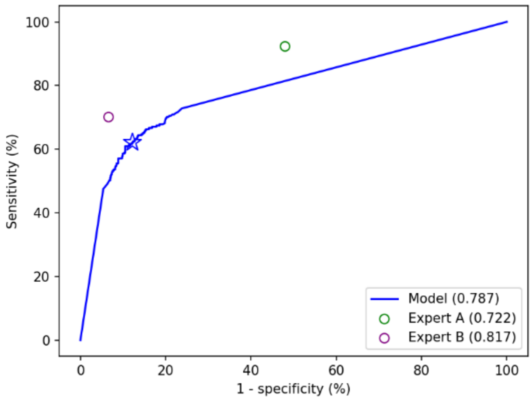

g

Supplementary Fig. 1 The ROC curves of the models trained and tested under various scanning conditions at the image level with two human experts' performance for comparison. a the ROC curve of the model tested with the images obtained by transducers of frequencies ≥14MHz. **b** the ROC curve of the model tested with the images obtained by transducers of frequencies <14MHz. **c** the ROC curve of the model tested with the images obtained in year 2019. **d** the ROC curve of the model tested with the images obtained in or before year 2018. **e** the ROC curve of the model tested with the images obtained by brand Supersonic. **f** the ROC curve of the model tested with the images obtained by brand Mindray. **g** the ROC curve of the model tested with the images obtained by the other brands. The blue star represents the performance of the model with the default threshold (0.5) to binarize outputs of the model. ‘ROC’, receiver operating characteristic.

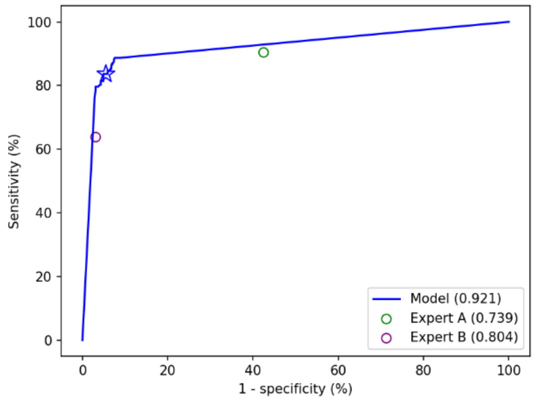
Sensitivity (%)
1 - specificity (%)
Model (0.921)
Expert A (0.739)
Expert B (0.804)

a

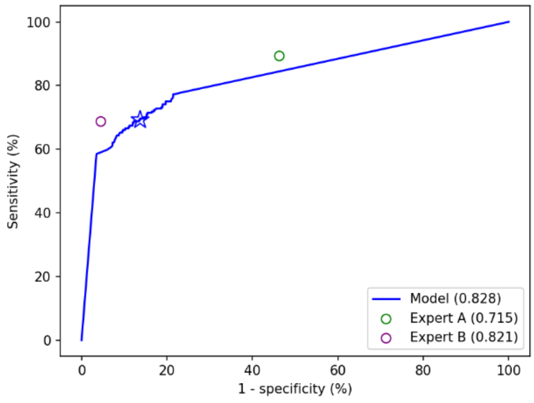
Sensitivity (%)
1 - specificity (%)
Model (0.828)
Expert A (0.715)
Expert B (0.821)

b

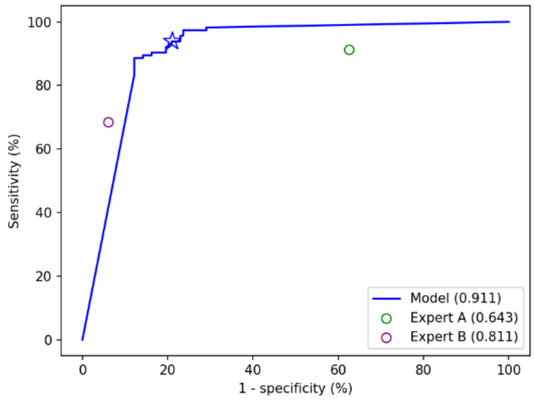
Sensitivity (%)
1 - specificity (%)
Model (0.911)
Expert A (0.643)
Expert B (0.811)

c

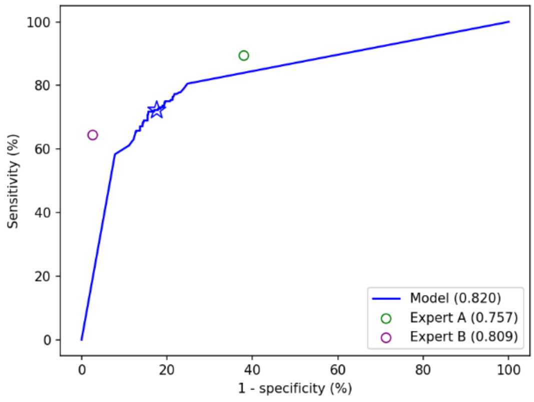
Sensitivity (%)
1 - specificity (%)
Model (0.820)
Expert A (0.757)
Expert B (0.809)

d

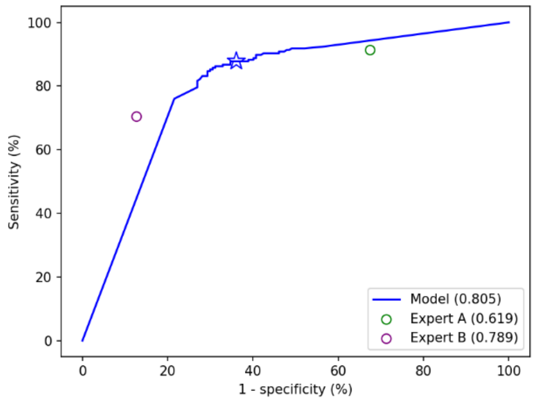
Sensitivity (%)
1 - specificity (%)
Model (0.805)
Expert A (0.619)
Expert B (0.789)

e

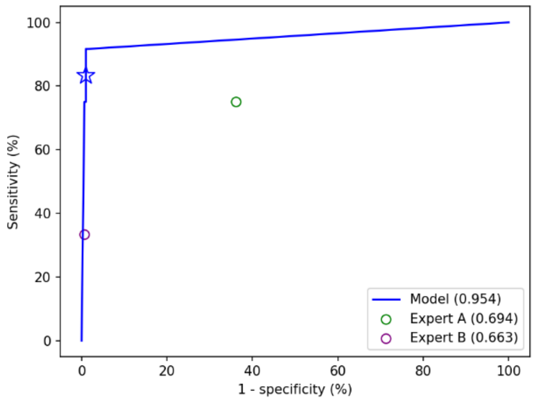
Sensitivity (%)
1 - specificity (%)
Model (0.954)
Expert A (0.694)
Expert B (0.663)

f

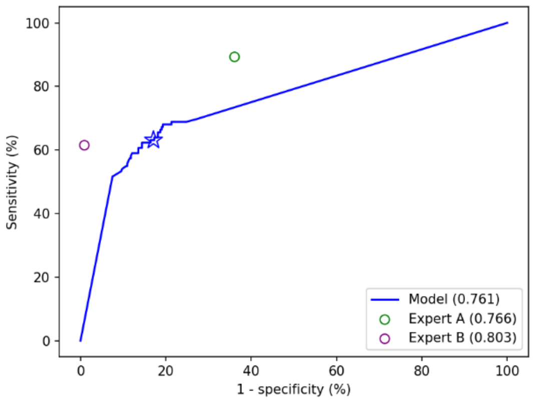

g

Supplementary Fig. 2 The ROC curves of the model trained and tested under various scanning conditions at the patient level with two human experts' performance for comparison. a the ROC curve of the model tested with the images obtained by transducers of frequencies ≥14MHz. **b** the ROC curve of the model tested with the images obtained by transducers of frequencies <14MHz. **c** the ROC curve of the model tested with the images obtained in year 2019. **d** the ROC curve of the model tested with the images obtained in or before year 2018. **e** the ROC curve of the model tested with the images obtained by brand Supersonic. **f** the ROC curve of the model tested with the images obtained by brand Mindray. **g** the ROC curve of the model tested with the images obtained by the other brands. The blue star represents the performance of the model with the default threshold (0.5) to binarize outputs of the model. 'ROC', receiver operating characteristic.

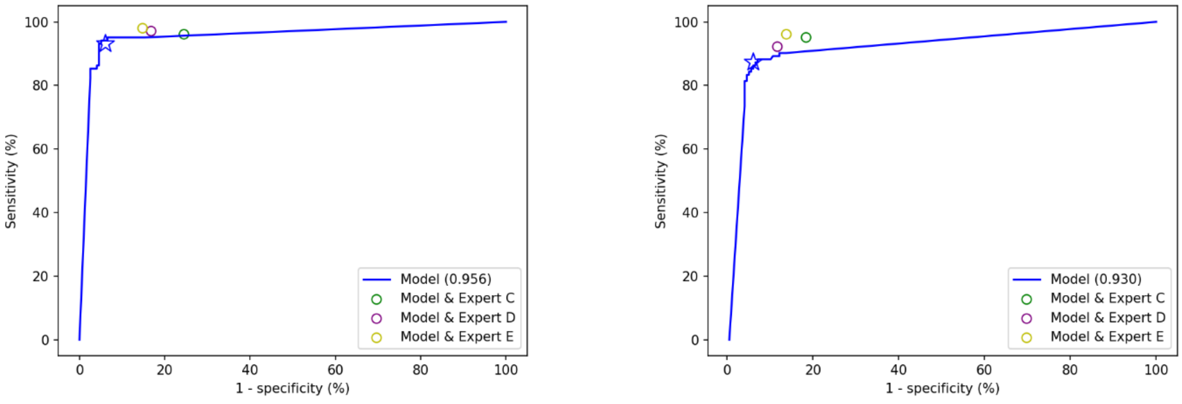

a b

Supplementary Fig. 3 The performance of the model and its combination with each human expert for the diagnosis of biliary atresia at the patient level on the external validation dataset, when the model made (a) majority vote diagnosis and (b) single-image diagnosis respectively at the patient-level.

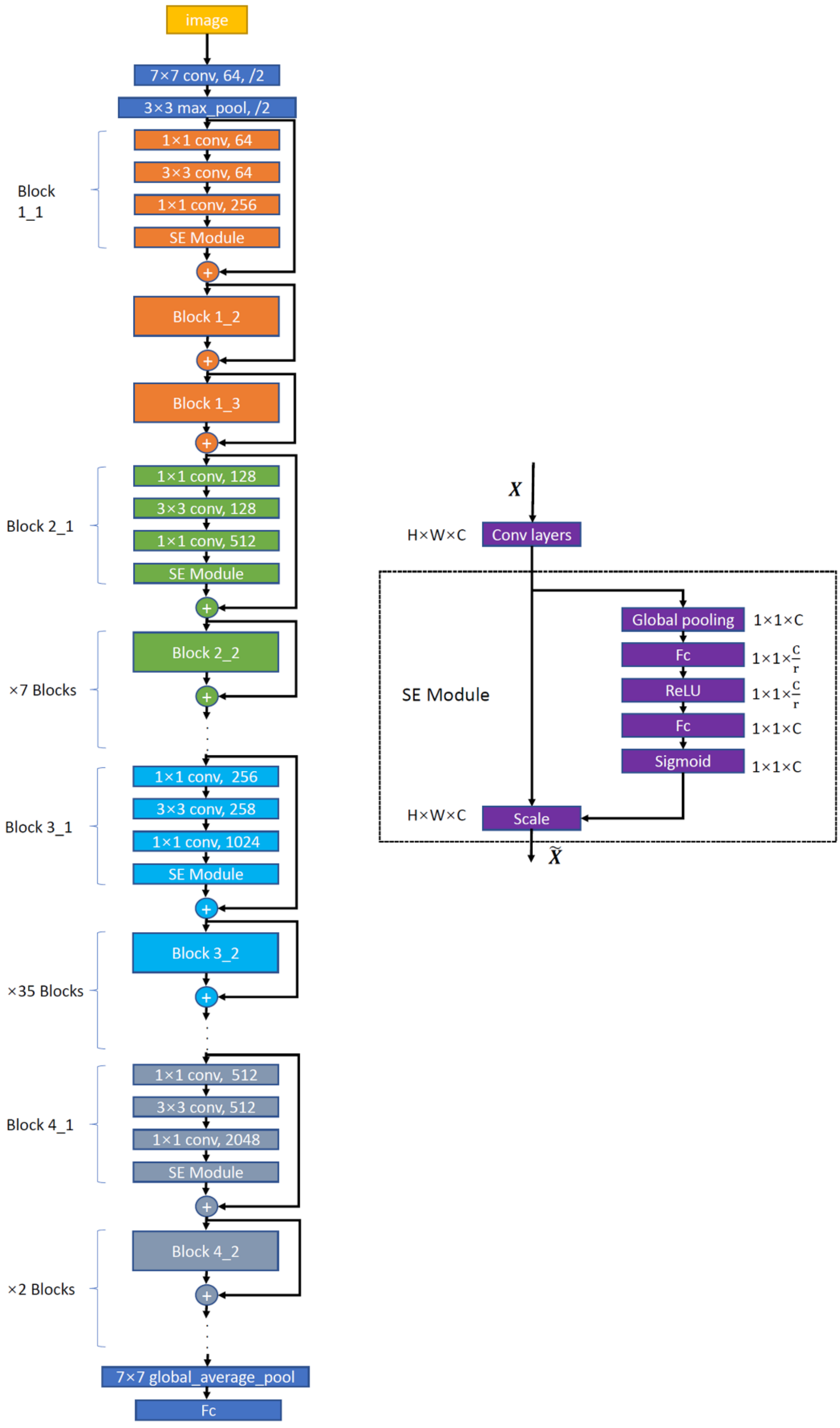

SE-ResNet

Supplementary Fig. 4 The SE-ResNet model structure.

References

1. Simonyan K, Zisserman A. Very Deep Convolutional Networks for Large-Scale Image Recognition. *arXiv* 1409.1556. (2014).

2. He K, Zhang X, Ren S, Sun J. Deep Residual Learning for Image Recognition.In: *Proceedings of the IEEE Conference on Computer Vision and Pattern Recognition (ICCV)*, 770-778 (2016).

3. Hu J, Shen L, Sun G. Squeeze-and-Excitation Networks. In: *Proceedings of the IEEE Conference on Computer Vision and Pattern Recognition (ICCV)*, 7132-7147 (2018).

4. Kukar M, Kononenko I. Cost-Sensitive Learning with Neural Networks. In: *European Conference on Artificial Intelligence (ECAI)*, 445-449 (1998).

5. Shin HC*, et al.* Deep Convolutional Neural Networks for Computer-Aided Detection: CNN Architectures, Dataset Characteristics and Transfer Learning. *IEEE Transactions on Medical Imaging* **35**, 1285-1298 (2016).

6. Srivastava N, Hinton G, Krizhevsky A, Sutskever I, Salakhutdinov RJTjomlr. Dropout: A Simple Way to Prevent Neural Networks from Overfitting. *Journal of Machine Learning Research* **15**, 1929-1958 (2014).
