# Supplementary material for: Ensembled deep learning model outperforms human experts in diagnosing biliary atresia from sonographic gallbladder images
Source: Nat Commun. 2021 Feb 24;12:1259. doi: 10.1038/s41467-021-21466-z (PMC7904842; doi:10.1038/s41467-021-21466-z)
Supplement: Supplementary file 3 — Reporting Summary [file 41467_2021_21466_MOESM3_ESM.pdf]

nature research

Corresponding author(s): Luyao Zhou

Last updated by author(s): Jan 18, 2021

Reporting Summary

Nature Research wishes to improve the reproducibility of the work that we publish. This form provides structure for consistency and transparency in reporting. For further information on Nature Research policies, see our Editorial Policies and the Editorial Policy Checklist.

Statistics

For all statistical analyses, confirm that the following items are present in the figure legend, table legend, main text, or Methods section.

n/a	Confirmed	
☐	☒	The exact sample size (*n*) for each experimental group/condition, given as a discrete number and unit of measurement
☐	☒	A statement on whether measurements were taken from distinct samples or whether the same sample was measured repeatedly
☐	☒	The statistical test(s) used AND whether they are one- or two-sided<br>*Only common tests should be described solely by name; describe more complex techniques in the Methods section.*
☒	☐	A description of all covariates tested
☐	☒	A description of any assumptions or corrections, such as tests of normality and adjustment for multiple comparisons
☐	☒	A full description of the statistical parameters including central tendency (e.g. means) or other basic estimates (e.g. regression coefficient) AND variation (e.g. standard deviation) or associated estimates of uncertainty (e.g. confidence intervals)
☐	☒	For null hypothesis testing, the test statistic (e.g. *F*, *t*, *r*) with confidence intervals, effect sizes, degrees of freedom and *P* value noted<br>*Give P values as exact values whenever suitable.*
☒	☐	For Bayesian analysis, information on the choice of priors and Markov chain Monte Carlo settings
☒	☐	For hierarchical and complex designs, identification of the appropriate level for tests and full reporting of outcomes
☒	☐	Estimates of effect sizes (e.g. Cohen's *d*, Pearson's *r*), indicating how they were calculated

Our web collection on statistics for biologists contains articles on many of the points above.

Software and code

Policy information about availability of computer code

Data collection	No software was used.
Data analysis	Software used for data analysis:SPSS software package version 25; MedCalc Statistical Software version 15.2.2 ; ImageJ version 1.52a ; PyTorch(1.5.1); torchvision(0.6.1) ; NumPy(1.17.0); scikit-learn(0.21.3); TensorboardX(1.8); PIL(7.1.2); tqdm(4.32.1); SimpleITK(1.2.0); pandas(0.25.0); Matplotlib(3.1.1); pretrained-models (0.7.4); and EfficientNet-PyTorch (0.6.3); The training code base for the deep learning framework is available at: https://github.com/youngyzzZ/Sonographic-Gallbladder-Images-for-BA-Diagnosis.

For manuscripts utilizing custom algorithms or software that are central to the research but not yet described in published literature, software must be made available to editors and reviewers. We strongly encourage code deposition in a community repository (e.g. GitHub). See the Nature Research guidelines for submitting code & software for further information.

Data

Policy information about availability of data

All manuscripts must include a data availability statement. This statement should provide the following information, where applicable:

- Accession codes, unique identifiers, or web links for publicly available datasets
- A list of figures that have associated raw data
- A description of any restrictions on data availability

Excel files containing raw data for Figures 1, 2, 3c and Tables 1-2 can be found in supplementary materials. Compressed images from the training dataset and external validation dataset are available in https://zenodo.org/record/4445734. All other datasets generated and analyzed in the current study (including original image data) are available from the corresponding author (L.Y.Z.) on reasonable request. Source data are provided with this paper.

Field-specific reporting

Please select the one below that is the best fit for your research. If you are not sure, read the appropriate sections before making your selection.

- [x] Life sciences
- [ ] Behavioural & social sciences
- [ ] Ecological, evolutionary & environmental sciences

For a reference copy of the document with all sections, see nature.com/documents/nr-reporting-summary-flat.pdf

Life sciences study design

All studies must disclose on these points even when the disclosure is negative.

Sample size	No sample-size calculation was performed; our work is a data-based deep learning study, thus, the more data we have, the better the outcomes it will be, so we are not sure whether our sample sizes are sufficient or not. The only thing we did was that we tried our best to obtain as many sonographic gallbladder images as possible from multiple hospitals.
Data exclusions	The exclusion criteria for patients are as follows: (1) the final diagnosis was unclear; (2) jaundice was caused by bile duct obstruction to which abdominal mass compression gave rise; (3) the patient had a history of abdominal surgery; (4) the visualization of gallbladder was indeterminate.
Replication	The ensembled deep learning model was verified and replicated using regular machine learning metrics on external validation dataset. The software of the model was released for replication on new data.
Randomization	Yes
Blinding	Yes.

Reporting for specific materials, systems and methods

We require information from authors about some types of materials, experimental systems and methods used in many studies. Here, indicate whether each material, system or method listed is relevant to your study. If you are not sure if a list item applies to your research, read the appropriate section before selecting a response.

Materials & experimental systems

n/a	Involved in the study	
x		Antibodies
x		Eukaryotic cell lines
x		Palaeontology and archaeology
x		Animals and other organisms
	x	Human research participants
	x	Clinical data
x		Dual use research of concern

Methods

n/a	Involved in the study	
x		ChIP-seq
x		Flow cytometry
x		MRI-based neuroimaging

Human research participants

Policy information about studies involving human research participants

Population characteristics	Infants younger than 5 months old with hyperbilirubinemia (serum direct bilirubin level >17.1 umol/L and the ratio of direct to total bilirubin level >20%) and suspected of BA were initially selected from 11 hospitals between January 2010 and June 2019;also, a number of infants who did not have any known liver diseases were randomly selected from the same 11 hospitals as non-BA. The patients were comprised of 714 boys and 427 girls with a median age of 57.0 days (IQR, 41.5–70.0 days) in the training cohort. The patients were comprised of 170 boys and 128 girls with a median age of 52.0 days (IQR, 32.8–65.0 days) in the external validation cohort.
Recruitment	The exclusion criteria for patients were as follows: (1) the final diagnosis was unclear; (2) jaundice was caused by bile duct obstruction to which abdominal mass compression gave rise; (3) the patient had a history of abdominal surgery; (4) the visualization of gallbladder was indeterminate. The exclusion criteria of (4) might cause selection bias that make the results too positive.
Ethics oversight	This study was approved by the institutional Clinical Research Ethics Committee of the First Affiliated Hospital of Sun Yat-sen University.

Note that full information on the approval of the study protocol must also be provided in the manuscript.

Clinical data

Policy information about clinical studies

All manuscripts should comply with the ICMJEguidelines for publication of clinical research and a completedCONSORT checklist must be included with all submissions.

Clinical trial registration	ChiCTR1800017428(www.chictr.org.cn).
Study protocol	The full trial protocol can be assessed at:http://www.chictr.org.cn/showproj.aspx?proj=29471.
Data collection	The data were collected from 11 different hospitals located in Chinese Mainland from 2010-2019.
Outcomes	The primary outcomes were pre-defined as the diagnostic performance of the ensembled deep learning model (sensitivity, specificity, negative predictive value, positive predictive value and accuracy); the secondary outcomes were the comparison of diagnostic performances between the ensembled deep learning model and human experts. All these measures were assessed by comparing them with the reference standards.
